# Skin infectome of patients with a tick bite history

**DOI:** 10.3389/fcimb.2023.1113992

**Published:** 2023-02-27

**Authors:** Jie Zhang, Yuan-Chun Zheng, Yan-Li Chu, Xiao-Ming Cui, Ran Wei, Cai Bian, Hong-Bo Liu, Nan-Nan Yao, Rui-Ruo Jiang, Qiu-Bo Huo, Ting-Ting Yuan, Jie Li, Lin Zhao, Lian-Feng Li, Qian Wang, Wei Wei, Jin-Guo Zhu, Mei-Chao Chen, Yan Gao, Fei Wang, Jin-Ling Ye, Ju-Liang Song, Jia-Fu Jiang, Tommy Tsan-Yuk Lam, Xue-Bing Ni, Na Jia

**Affiliations:** ^1^ State Key Laboratory of Pathogen and Biosecurity, Beijing Institute of Microbiology and Epidemiology, Beijing, China; ^2^ Department of Cardiology, Mudanjiang Forestry Central Hospital, Mudanjiang, China; ^3^ The Affiliated Hospital of Shandong University of Traditional Chinese Medicine, Jinan, China; ^4^ Department of Infectious Diseases Control and Prevention, Chinese People's Liberation Army of China (PLA) Center for Disease Control and Prevention, Beijing, China; ^5^ Institute of Nuclear, Biological, and Chemical weapons (NBC) Defence, PLA Army, Beijing, China; ^6^ School of Medicine, Nankai University, Tianjin, China; ^7^ Institute of EcoHealth, School of Public Health, Cheeloo College of Medicine, Shandong University, Jinan, Shandong, China; ^8^ Department of Health Quarantine, ManZhouLi Customs District, Manzhouli, China; ^9^ State Key Laboratory of Emerging Infectious Diseases and Centre of Influenza Research, School of Public Health, The University of Hong Kong, Pok Fu Lam, Hong Kong SAR, China

**Keywords:** ticks, skin, patients, infectome, metatranscriptomic sequencing

## Abstract

**Introduction:**

Ticks are the most important obligate blood-feeding vectors of human pathogens. With the advance of high-throughput sequencing, more and more bacterial community and virome in tick has been reported, which seems to pose a great threat to people.

**Methods:**

A total of 14 skin specimens collected from tick-bite patients with mild to severe symptoms were analyzed through meta-transcriptomic sequencings.

**Results:**

Four bacteria genera were both detected in the skins and ticks, including Pseudomonas, Acinetobacter, Corynebacterium and Propionibacterium, and three tick-associated viruses, Jingmen tick virus (JMTV), Bole tick virus 4 (BLTV4) and Deer tick mononegavirales-like virus (DTMV) were identified in the skin samples. Except of known pathogens such as pathogenic rickettsia, Coxiella burnetii and JMTV, we suggest Roseomonas cervicalis and BLTV4 as potential new agents amplified in the skins and then disseminated into the blood. As early as 1 day after a tick-bite, these pathogens can transmit to skins and at most four ones can co-infect in skins.

**Discussion:**

Advances in sequencing technologies have revealed that the diversity of tick microbiome and virome goes far beyond our previous understanding. This report not only identifies three new potential pathogens in humans but also shows that the skin barrier is vital in preventing horizontal transmissions of tick-associated bacteria or virus communities to the host. It is the first research on patients’ skin infectome after a tick bite and demonstrates that more attention should be paid to the cutaneous response to prevent tick-borne illness.

## Introduction

Ticks are the most important vectors of human pathogens, causing increasing public health burdens worldwide. Ticks can cause a variety of diseases, such as tick-borne viral illness, Lyme disease, spotted fever group rickettsiosis, human anaplasmosis, and human babesiosis (Jia et al., 2020b). Ticks transmit infectious agents through continuous attachment to the host’s skin and blood feeding for days. The skin plays a key role in vector-borne diseases because it is the site where the arthropod co-inoculates pathogens and its saliva. It is becoming increasingly evident that the skin is not simply a physical barrier, but is also an organ important in innate immunity, as the first barrier met by the tick and the pathogen (Wikel, 2013; Boulanger and Wikel, 2021). The cutaneous response contributes to the tick-borne pathogen multiplying and sometimes persisting before disseminating, as in Lyme borreliosis (Bernard et al., 2020), spotted fever rickettsiosis (Heinze et al., 2014; Jia et al., 2020a), and Jingmen tick virus infection (Jia et al., 2019). Although various pathogens have been detected in skin lesions, the skin infectome after a tick bite remains largely unknown. This knowledge will undoubtedly provide a deep understanding of the risks presented by tick bites in humans.

In recent years, next-generation sequencing has been successfully used to characterize the complete tick microbiome, identify novel and unexpected pathogens, and elucidate tick microbiome–host interactions, with the goal of developing antivectorial control measures (Carpi et al., 2011; Hawlena et al., 2013; Nakao et al., 2013; Williams-Newkirk et al., 2014). We collected 14 skin lesions from patients in a sentinel hospital who had a history of a tick bite. The microbiome and virome of skin biopsy specimens were identified utilizing a meta-transcriptomics approach, and pathogens that could potentially be transmitted from skin to blood were analyzed, providing valuable data illustrating the horizontal transmission of pathogens from ticks to humans.

## Materials and methods

### Sample collection

Patients who had been bitten by ticks and sought medical treatment at Mudanjiang Forestry Central Hospital in the Heilongjiang Province of China were included in this study. Ticks were aseptically removed from the patient’s body, and an area of skin tissue measuring 1 cm × 1 cm was excised from the site of the tick bite in a sterile operation conducted by hospital surgeons, and then stored in RNase-free microfuge tubes at –80°C. Venous blood samples of volume 2 mL were collected into vacuum blood collection tubes. The medical information of each patient was recorded with their consent.

### Ethics approvals

All participants provided written informed consent for this study, which was approved by the Mudanjiang Forestry Central Hospital Review Board and the Academy of Military Medical Sciences Review Board, China.

### Sample preparation and sequencing

Total RNA extraction was performed using an AllPrep DNA/RNA Mini Kit (lot no. 80204; Qiagen, USA), with modifications. Briefly, tick and skin samples were quickly thawed and homogenized in an RLT solution in liquid nitrogen. The tick homogenate, skin homogenate, and blood samples were incubated at 55°C for 10 min with proteinase K (Qiagen) and centrifuged for 30 s at 12,000 × *g*. The homogenized lysate was transferred to an AllPrep DNA spin column and centrifuged for 30s at 8,000 × *g*. The flowthrough and the AllPrep RNA spin column were used for further RNA purification, in accordance with the manufacturer’s instructions.

The extracted RNA was used for transcriptome sequencing (RNA-seq) after RNA quantification and qualification (Thermo Scientific NanoDrop2000). The rRNA was removed using RiBo-Zero Gold rRNA Removal Reagents (human/mouse/rat) (Illumina), then the sequencing library was prepared following Illumina’s standard protocol (Modi et al., 2021). Paired-end (2 × 150-bp) sequencing of the RNA library was performed on an Illumina HiSeq 4000 platform at Novogene Tech (Beijing, China).

### Discovery and assembly of viral genomes

RNA sequencing reads were assembled *de novo* using the Trinity program (Grabherr et al., 2011). Assembled contigs were subject to Basic Local Alignment Search Tool—Nucleotide (BLASTn) analysis and compared with a non-redundant nucleotide (nt) database and to BLASTx analysis and compared with all non-redundant protein (nr) databases downloaded from GenBank, and the threshold *E*-value was set to 1e^–5^. The contigs that best matched the virus were kept for downstream analysis. Putative viral contigs were further merged by high-identity overlaps using the SeqMan program of Lasergene package v.7.1 (DNASTAR, Madison, WI, USA). Original reads were aligned to the contigs again using Bowtie2 (Langmead and Salzberg, 2012) to close the remaining gaps and verify the assembly in the Integrated Genomics Viewer (Thorvaldsdóttir et al., 2013).

### Bacterial taxonomic classification and read counts estimation

All host reads were first removed by mapping against *Homo sapiens* (NW_012132914.1) using Bowtie2 (Langmead and Salzberg, 2012). The remaining reads were analyzed against the SILVA 16s rRNA database (https://www.arb-silva.de/) using BLASTn with a threshold *E*-value of < 1e–5. After annotation, the Ribosome Database Project (RDP) taxonomic database was selected for taxonomic assignment. The bacterial read counts were summarized at genus level, and histograms were plotted to visualize the results. The contigs of pathogenic bacteria were identified through BLASTn analysis based on the criterion that more than 80% of base pairs aligned to the reference sequence with similarity higher than 85% (nucleotides) or 90% (amino acids). Read counts were estimated by mapping reads to the reference genome of the same species as classified above using Bowtie2 (Langmead and Salzberg, 2012).

### Phylogenetic analyses

Nucleotide alignments were prepared with the reference sequences at the species level for pathogenic bacteria based on the 16s rRNA gene and for the virus based on the RNA-dependent RNA polymerase (*RdRp*) gene using the E-INS-i algorithm in MAFFT (multiple alignment using fast Fourier transformation), version 7 (Katoh and Standley, 2013). Ambiguously aligned regions were removed using trimAl (Capella-Gutiérrez et al., 2009). The GTR+I+G model was identified to be the best-fit nucleotide substitution model using ProtTest 3.4 (Darriba et al., 2011), which was used for maximum likelihood (ML) phylogeny reconstruction with bootstrap tests (1,000 replicates) in PhyML v.3.0 (Guindon and Gascuel, 2003). The ML trees were visualized with FigTree v.1.4.2.

### Kyoto Encyclopedia of Genes and Genomes pathway analyses

The above filtered non-host reads were subject to GhostKOALA (https://www.kegg.jp/ghostkoala/) against both the prokaryotic Kyoto Encyclopedia of Genes and Genomes (KEGG) genes database at the genus level and the eukaryotic KEGG genes database at the family level using CD-HIT (Cluster Database at High Identity with Tolerance) clusters with 50% identity cut-off value.

## Results

A total of 14 patients with a tick bite were enrolled in this study. Nine patients saw a doctor while the tick was still on their bodies (four *Ixodes persulcatus*, four *Dermacentor silvarum*, and one *Haemaphysalis concinna*). The ticks were most likely to be found on the scalp (4/9) or the back (2/9). The interval between the tick biting and sample collection ranged from 1 to 30 days, with a median of 8 days. The necrotic cells were distributed in the epidermis of eschars, while inflammatory infiltration by lymphocytes was identified in some skin lesions by hematoxylin and eosin (HE) staining (**Figure S1**). The clinical symptoms varied from mild to severe and included local itching, headache, rash, lymphadenectasis, and fever, with laboratory tests showing that some patients had an abnormal liver function and neutropenia (**Table 1**).

**Table 1:** The tick bite history and clinical manifestation of patients enrolled in the study.

Patient	Tick on patients/engorgement	HE staining of skin lesions	Clinical symptoms/laboratory tests	Interval from tick bite to skin sample collection (days)	Detected pathogens in skin or in blood
Case 1	*Ixodes persulcatus*/no engorgement	NA	No	1	JMTV	
Case 2	*Dermacentor silvarum*/full engorgement	Necrosis	Local swollen	10	JMTV	
Case 3	*I. persulcatus*/half engorgement	Inflammatory infiltration	No	NA	JMTV	
Case 4	*I. persulcatus*/half engorgement	NA	No	2	JMTV, CRT, *Roseomonas cervicalis*, DTMV	
Case 5	*D. silvarum/*full engorgement	NA	Itching	3	JMTV, *Rickettsia raoultii*	
Case 6	*Haemaphysalis concinna*/no engorgement	NA	No	1	JMTV, CRT	
Case 7	*D. silvarum/*full engorgement	Necrosis	Lymphadenectasis	30	JMTV, *Roseomonas cervicalis*, BLTV4	
Case 8	*I. persulcatus*/half engorgement	Inflammatory infiltration	Headache/neutropenia	8	JMTV*, Coxiella burnetii*	
Case 9	*D. silvarum/*full engorgement	Necrosis	Lymphadenitis	15	JMTV*, Rickettsia raoultii*	
Case 10	NA	Necrosis	Itching, eschar (hospitalization)/ALT and AST elevation	10	JMTV*, Coxiella burnetii*, BLTV4	
Case 11	NA	Inflammatory infiltration	Eschar	5	*Rickettsia raoultii*	
Case 12	NA	Inflammatory infiltration	Fever, rash (hospitalization)/ALT elevation	9	No	
Case 13	NA	Inflammatory infiltration	Fever, rash lymphadenitis/AST elevation	14	*Rickettsia sibirica*	
Case 14	NA	Normal	No	1	No	

NA, not available; CRT, Candidatus Rickettsia tarasevichiae; JMTV, Jingmen tick virus; DTMV, deer tick Mononegavirales-like virus; BLTV4, Bole tick virus 4; ALT, alanine aminotransferase; AST, aspartate aminotransferase; HE, hematoxylin and eosin.

### Skin bacterial community and blood dissemination

**Figure 1:**
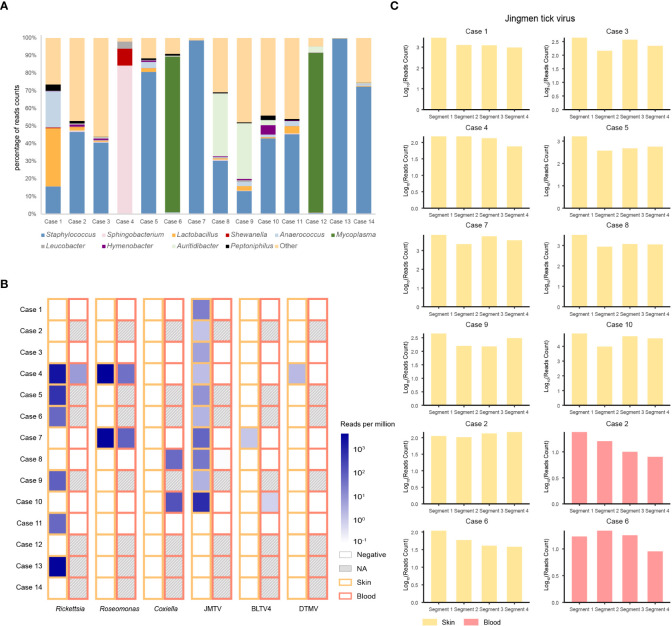
Bacteria and viruses identified in skin samples from 14 patients with a tick bite as determined by metatranscriptomic sequencing. **(A)** Percentage of bacterial read counts in the skin samples after a tick bite. **(B)** Comparisons of the bacterial and viral read counts of six tick-borne pathogens in skin and blood samples. **(C)** Viral abundance of the Jingmen tick virus by viral segments in the positive skin and blood samples.

The predominant bacterial genus isolated from the skin around a tick bite was in most cases *Staphylococcus*, but in four cases (i.e., cases 1, 4, 6, and 12) it was *Lactobacillus*, *Sphigobacterium*, or *Mycoplasma* (**Figure 1A**). Examining the microbiome in ticks detached from the patients (**Figure S2**) revealed that four bacterial genera were common to the skin and tick: *Pseudomonas*, *Acinetobacter*, *Corynebacterium*, and *Propionibacterium*.

**Figure 2:**
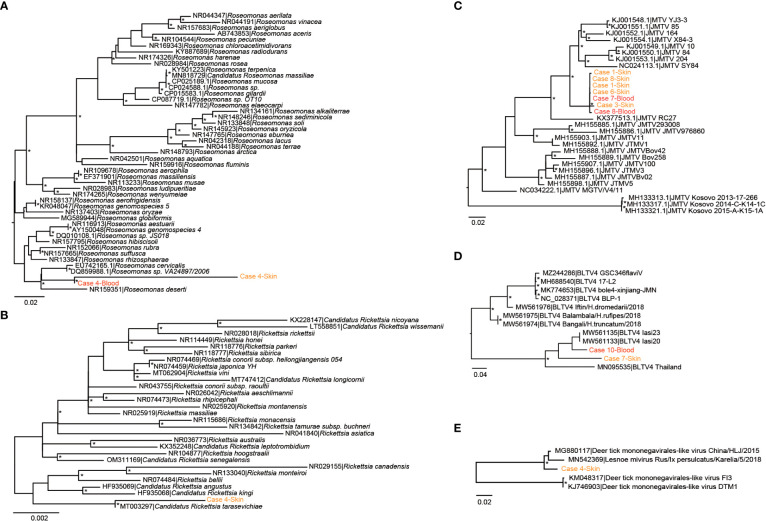
Phylogenetic analyses of (potential) pathogens detected in patients in this study. **(A)** *Roseomonas*, **(B)** *Rickettsia*, **(C)** Jingmen tick virus, **(D)** Bole tick virus 4, and **(E)** deer tick *Mononegavirales*-like virus. The symbol * mean the value of bootstrap for this branch >70.

We then focused on pathogenic bacteria and found that *Coxiella burnetii*, *Candidatus Rickettsia tarasevichiae* (CRT), *Rickettsia raoultii*, *Rickettsia sibirica*, and *Roseomonas cervicalis* could be detected among tick bite patients’ skin and blood samples by RNA-seq (**Table 1**, **Figure 1B**). The 16s rRNA gene sequences of the above pathogens showed, respectively, 87.3%–87.5%, 93.8%–99.9%, 92.5%–98.2%, 99.7%–100%, and 88.5%–90.0% Nucleotide (nt) similarity with the reference strains (**Table S1**). Interestingly, *Roseomonas* could be identified in both skin and blood, whereas *Coxiella* was detected only in blood samples (cases 8 and 10; **Figure 1B**). Bacterial contigs were successfully assembled from case 4, who was co-infected with CRT and *R. cervicalis*, and were used for constructing phylogenic trees. The sequences fell into the clade with *R. cervicalis*, or with CRT, respectively (**Figure 2**). The rickettsial pathogens could be horizontally transmitted from ticks to skin as early as 1 day after a tick bite and could be amplified for at least 15 days. The interval between tick bite and skin lesion collection was 2–30 days for *R. cervicalis* infections (**Table 1**).

The KEGG pathways of the microbiomes on patients’ skin and blood were analysed to understand the microbiome response. In general, microbial communities in the skin showed higher activity than those in the blood, owing to the fact most patients did not have bacteremia. The metabolic pathways of the microbiome were mostly activated, leading to the biosynthesis of secondary metabolites and microbial metabolism in diverse environments. However, the cofactor and vitamin metabolic pathways, as well as the immune system of the microbiome, were activated to a lesser extent.

### Skin virome and blood dissemination

Three viruses, Jingmen tick virus (JMTV), Bole tick virus 4 (BLTV4), and deer tick *Mononegavirales*-like virus (DTMV), could be detected in the skin, whereas JMTV and BLTV4 could also be detected in blood (**Figure 1B**).

Segment 1 of JMTV, encoding the NS5-like protein with the conserved motifs for methyltransferase and RNA-dependent RNA polymerase, was found in nine skin samples and was the most abundant segment, followed by segment 3 of NS2b–NS3-like protein, which relates to the serine protease domain and helicase domain, and was found in seven patients (**Figure 1C**). Segments 2 and 4, which encode structural proteins, were less abundant (**Figure 1C**). The viral abundance of segments of JMTV varied between two blood samples (**Figure 1C**). JMTV isolated from patients showed 94.9%–100% nt similarity in the *RdRp* gene, with ticks collected in China, and formed a separate clade with other strains detected from ticks (**Table S1**, **Figure 2**). BLTV4 belongs to the unclassified *Riboviria*, and has not previously been reported in skin or blood from patients with a tick bite history. BLTV4 in the skin (case 7) and in the blood (case 10) showed 92.4% and 90.9% nt similarity, respectively, to strains previously identified from *Dermacentor reticulatus* in Romania (**Table S1**, **Figure 2**). DTMV is an unclassified virus and has not yet been reported in samples from patients. DTMV was detected from case 4’s skin sample, with an abundance of 257 reads, showing 99.3% nt similarity to DTMV isolate China/HLJ/2015 (**Table S1**, **Figure 2**). This reference strain in the phylogenic tree was identified in *I. persulcatus* from Heilongjiang province in China, where our patients were living.

We noticed that JMTV and DTMV could be transmitted to patients’ skin as soon as 1 or 2 days after a tick bite, and BLTV4 could be detected in the skin until 30 days after a tick bite (**Table 1**). Co-infections with tick-borne viruses and bacteria in the skin were found in half of the patients, and up to four pathogens could be detected in one skin lesion specimen.

## Discussion

Advances in sequencing technologies have revealed that the diversity of tick microbiome and virome goes far beyond our previous understanding (Li et al., 2021; Narasimhan et al., 2021); it is, therefore, interesting to characterize the host skin infectome after a tick feeds. Transcriptome analysis of skin lesions in patients with a clear tick bite history revealed that four bacterial genera, *Pseudomonas*, *Acinetobacter*, *Corynebacterium*, and *Propionibacterium*, were common in ticks and patients’ skin. However, we cannot determine whether the detected bacteria were in the tick or on the tick, or whether pathogens or bacteria were transmitted from the tick’s surface. In addition, excluding known bacterial pathogens, such as the spotted fever group rickettsia *C. burnetii* (Huang et al., 2021; Fu et al., 2022), a potentially new pathogen, *R. cervicalis*, was detected in both the blood and skin samples of patients. Although more than 10 viral families were found in ticks by RNA-seq in our previous study (Li et al., 2021), only three viruses (i.e., JMTV, BLTV4, and DTMV) could be detected in skin biopsy specimens. This report not only identifies three new potential pathogens in humans but also shows that the skin barrier is vital in preventing horizontal transmissions of tick-associated bacteria or virus communities to the host.

The co-transmission of vector microbiomes and vector-borne pathogens has been previously suspected in insects (Finney et al., 2015). However, regarding the transmission of tick-borne pathogens, very few studies have been performed to investigate the potential transmission of microbiota during the process of a tick bite. In addition, very few studies have investigated the role of skin microbiota in pathogen transmission at the skin interface. At the skin interface, the microbiome also contributes to the regulation of inflammation and is likely to affect the host’s responses to the tick bite. The skin might play a key role in the process of tolerance as the first interface met by the tick and the pathogen (Bernard et al., 2020). Our study also implies that the duration of tick blood feeding is important for the successful passage of an infectious agent into the bite site and the subsequent establishment of infection, although a larger sample size is required to confirm our results. Furthermore, this observation has practical implications for disease prevention.

*Rosemonas* is thought to be opportunistically pathogenic to humans, particularly immunocompromised patients with underlying diseases such as acute leukemia, cancer, or rheumatoid arthritis (Dé et al., 2004). A previous study found that *Rosemonas* sp. (*R. cervicalis*) could be isolated from adult *Dermacentor nuttalli* and its larval progeny, and obtained evidence of transovarial transmission (Liu et al., 2010). This study further demonstrates the role of the tick in transmitting *R. cervicalis* to humans.

Tick-associated DTMV was also detected in the skin of case 4, but not from the same patient’s blood sample. It showed high similarity with DTMV strain China/HLJ/2015, which was identified in *I. persulcatus* in the same province as our patients, and with Lesnoe mivirus, which is from the same tick species but is found in Russia. Another horizontally transmitted tick virus is BLTV4. It is particularly noteworthy that we detected BLTV4 in both skin and blood samples from two patients. BLTV4 was first identified in a metaviromic study carried out in China in 2012 and was subsequently reported in *Hyalomma asiaticum*, *Hyalomma truncatum*, *Dermacentor reticulatus*, and *Rhipicephalus microplus* collected in Romania and Kenya (Shi et al., 2016; Zhang et al., 2021). We cannot provide evidence of the pathogenicity of these viruses; however, considering their wide distribution in terms of both tick species and geographic location, we stress the importance of continuous active surveillance of patients with a tick bite history.

JMTV was first identified in *R. microplus* ticks sampled from Jingmen in Hubei Province and Wenzhou in Zhejiang Province, China, in 2010 (Qin et al., 2014). The genome of JMTV comprises four segments of single-stranded positive-sense RNA. Two segments exhibit homology with the non-structural protein NS3 and NS5 sequences of flaviviruses, whereas the other two segments are unique and have no known homologs (Qin et al., 2014). Since its initial identification, JMTV has been identified in several regions of China as well as in Brazil, Kosovo, Uganda, Turkey, the French Antilles, and Trinidad and Tobago (Ladner et al., 2016; Emmerich et al., 2018; Souza et al., 2018; Dinçer et al., 2019; Pascoal et al., 2019; Sameroff et al., 2019; Temmam et al., 2019; Guo et al., 2020). JMTV can infect a broad range of animal hosts, including cattle, rodents, bats, and primates (Souza et al., 2018; Sameroff et al., 2019; Guo et al., 2020). It has been reported as the causative agent of febrile disease in China and Kosovo (Emmerich et al., 2018; Jia et al., 2019). In our study, among 10 patients with JMTV infection, seven had been co-infected with either bacteria or other viruses in the skin. More in-depth laboratory studies and long-term epidemiological studies are needed to clarify the pathogenicity of JMTV in humans.

We found that some pathogenic bacteria could infest patients very quickly after a tick bite, as exemplified by CRT and *R. cervicalis* (e.g., cases 4 and 6). However, it appears that viruses took several days (i.e., 8–30 days) to successfully cause viremia (e.g., JMTV and BLTV4 in cases 7, 8, and 10). This may be related to the difference in the abundance of bacteria and viruses in ticks, as well as to differences in humans’ immune responses to bacteria and viruses. These are possible directions for future study.

This work is limited by the small sample size. In addition, the pathogenicity of new agents deserves further verification. The detection of seroconversion or a fourfold increase in antibodies in reaction to these bacteria and viruses would be useful to clarify their pathogenicity. Nevertheless, this work is the first research on patients’ skin infectome after a tick bite and demonstrates that more attention should be paid to the cutaneous response to prevent tick-borne illness.

## Data availability statement

The data presented in the study are deposited in the Gene Expression Omnibus data repository, accession number GSE141235.

## Ethics statement

The studies involving human participants were reviewed and approved by Mudanjiang Forestry Central Hospital. The patients/participants provided their written informed consent to participate in this study.

## Author contributions

NJ contributed to the conception and design of the study. XBN performed the statistical analysis. NJ, X-BN, and T-YL reviewed the draft. JZ wrote the first draft of the manuscript. X-MC, R-RJ, and RW investigated the hospital. X-MC, J-FJ, J-LS, J-LY, FW, YG, M-CC, J-GZ, WW, JL, T-TY, Q-BH, N-NY, CB, Y-LC, and Y-CZ collected the samples. H-BL and LZ contributed to the methodology. L-FL organized the database. QW contributed to the data curation. All authors contributed to the article and approved the submitted version.
