# Supplementary material for: Skin infectome of patients with a tick bite history
Source: Front Cell Infect Microbiol. 2023 Feb 27;13:1113992. doi: 10.3389/fcimb.2023.1113992 (PMC10008932; doi:10.3389/fcimb.2023.1113992)

**Supplementary material**

**TABLE S1** Best match of blast results for tick-borne pathogen contigs.

**FIGURE S1** HE staining of skin samples from tick-bite patients.


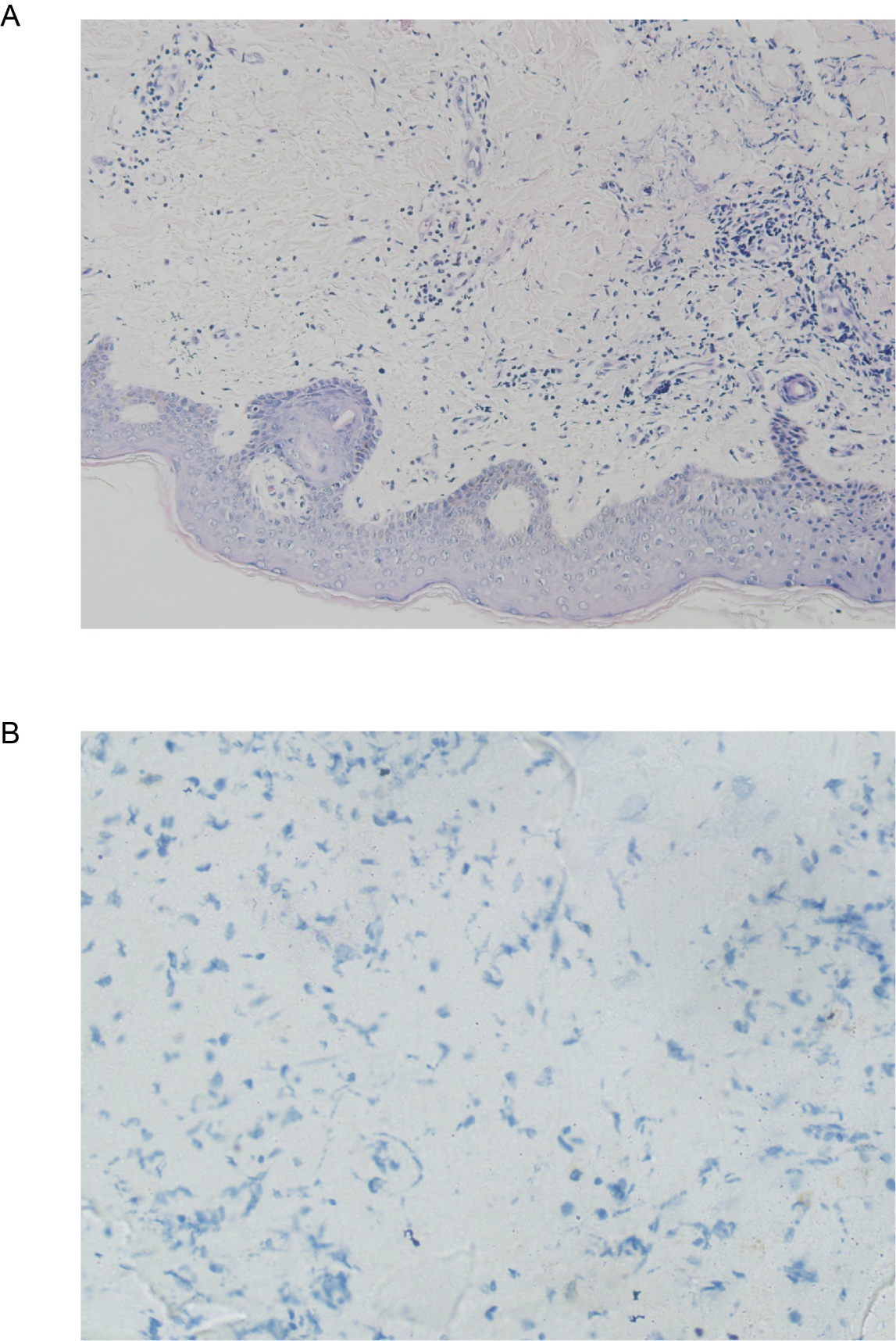


**FIGURE S2** Bacteria composition of tick samples from patients. Nine ticks were collected but only seven were successfully RNA-sequencing.


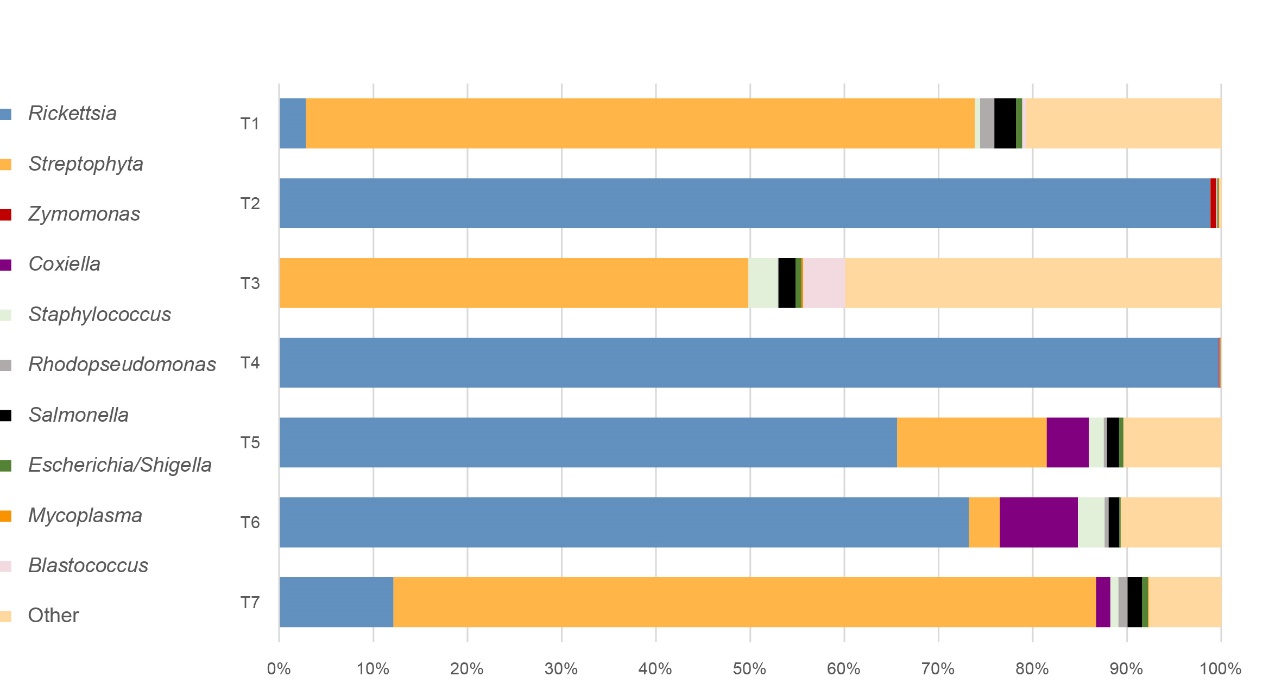


**FIGURE S3** Microbial pathways expressed by the bacteria in skin and blood samples through KEGG analyses.


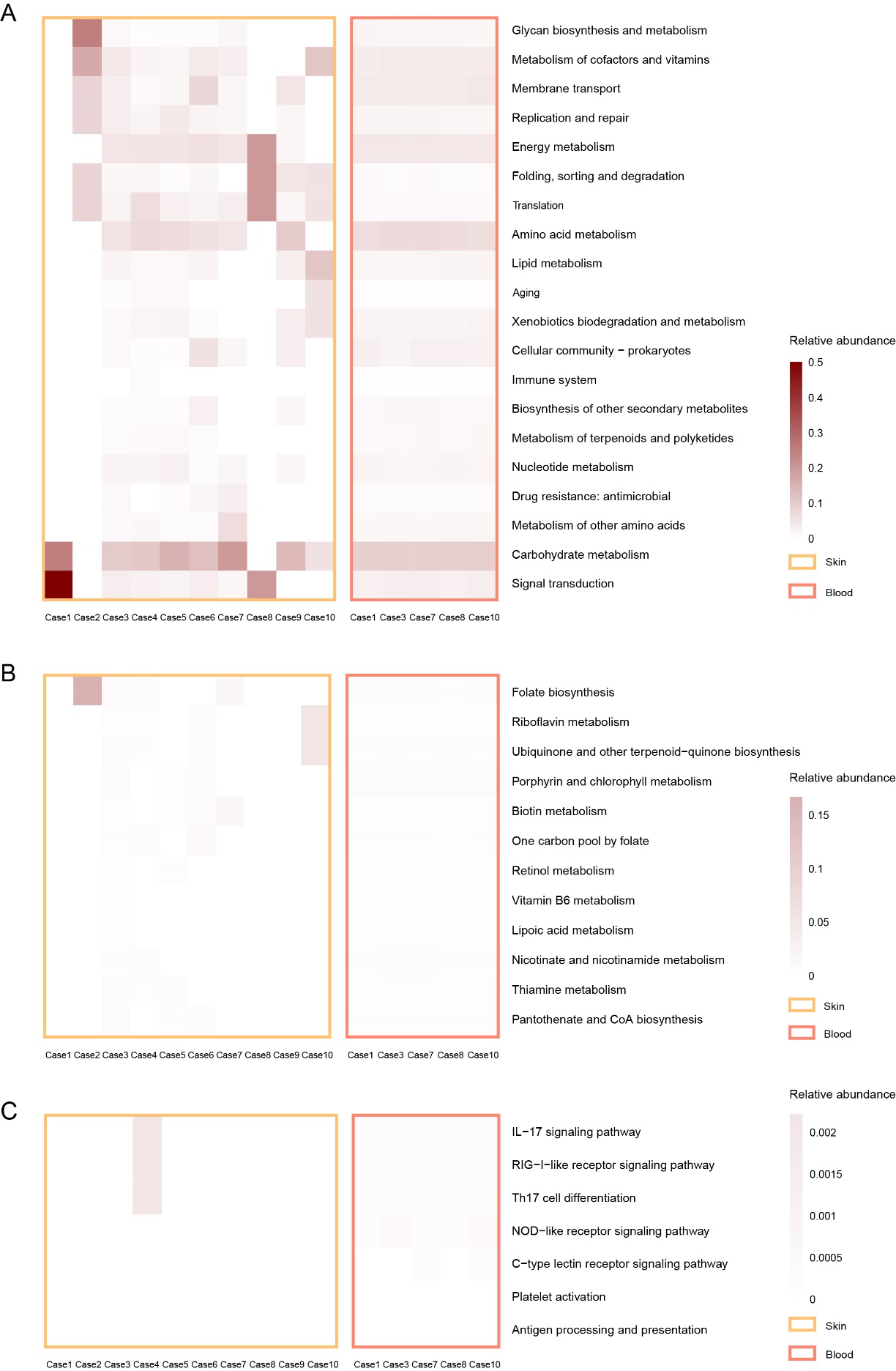

Supplement: Supplementary file 1 [file Table_1.docx]
